# Refueling the post COVID-19 brain: potential role of ketogenic medium chain triglyceride supplementation: an hypothesis

**DOI:** 10.3389/fnut.2023.1126534

**Published:** 2023-06-21

**Authors:** Angela G. Juby, Stephen C. Cunnane, Diana R. Mager

**Affiliations:** ^1^Division of Geriatrics, Department of Medicine, University of Alberta, Edmonton, AB, Canada; ^2^Research Center on Aging, Université de Sherbrooke, Sherbrooke, QC, Canada; ^3^Agriculture Food and Nutrition Science, University of Alberta, Edmonton, AB, Canada

**Keywords:** post (long) COVID-19, subjective cognitive decline, beta-hydroxybutyrate, medium chain triglyceride, brain fog

## Abstract

COVID-19 infection causes cognitive changes in the acute phase, but also after apparent recovery. Over fifty post (long)-COVID symptoms are described, including cognitive dysfunction (“brain fog”) precluding return to pre-COVID level of function, with rates twice as high in females. Additionally, the predominant demographic affected by these symptoms is younger and still in the workforce. Lack of ability to work, even for six months, has significant socio-economic consequences. This cognitive dysfunction is associated with impaired cerebral glucose metabolism, assessed using ^18^F-fluorodeoxyglucose-positron emission tomography (FDG-PET), showing brain regions that are abnormal compared to age and sex matched controls. In other cognitive conditions such as Alzheimer’s disease (AD), typical patterns of cerebral glucose hypometabolism, frontal hypometabolism and cerebellar hypermetabolism are common. Similar FDG-PET changes have also been observed in post-COVID-19, raising the possibility of a similar etiology. Ketone bodies (B-hydroxybutyrate, acetoacetate and acetone) are produced *endogenously* with very low carbohydrate intake or fasting. They improve brain energy metabolism in the face of cerebral glucose hypometabolism in other conditions [mild cognitive impairment (MCI) and AD]. Long-term low carbohydrate intake or prolonged fasting is not usually feasible. Medium chain triglyceride (MCT) is an *exogenous* route to nutritional ketosis. Research has supported their efficacy in managing intractable seizures, and cognitive impairment in MCI and AD. We hypothesize that cerebral glucose hypometabolism associated with post COVID-19 infection can be mitigated with MCT supplementation, with the prediction that cognitive function would also improve. Although there is some suggestion that post COVID-19 cognitive symptoms may diminish over time, in many individuals this may take more than six months. If MCT supplementation is able to speed the cognitive recovery, this will impact importantly on quality of life. MCT is readily available and, compared to pharmaceutical interventions, is cost-effective. Research shows general tolerability with dose titration. MCT is a component of enteral and parenteral nutrition supplements, including in pediatrics, so has a long record of safety in vulnerable populations. It is not associated with weight gain or adverse changes in lipid profiles. This hypothesis serves to encourage the development of clinical trials evaluating the impact of MCT supplementation on the duration and severity of post COVID-19 cognitive symptoms.

## Introduction/background

Current statistics^1^ report that the SARS-CoV-2 pandemic (COVID-19) has exceeded 650 million cases worldwide, with over 6 million reported deaths. This likely underestimates the numbers as it does not capture cases from countries where diagnostic testing is not readily available, or untested cases with more mild disease. Worldwide new variants keep emerging, and there is persistent infection and re-infection.

Following apparent recovery from COVID-19 infection, several persistent post-COVID-19 symptoms have been documented in a cohort of patients. These post COVID-19 symptoms were first reported in the media (1, 2), and data now suggest one in three people are not fully recovered after several weeks (3), with as many as fifty symptoms being described (4). They include persistent exercise intolerance, breathlessness, cough, anxiety, palpitations, poor concentration, intense fatigue, mood swings, muscle/joint pains, headaches, attention disorder and memory loss or ‘brain fog’ (5). Because of the increasing number of cases of acute and long COVID worldwide, in October 2021 the World Health Organization clearly defined long COVID as a condition that “occurs in individuals with a history of probable or biologically confirmed SARS-COV-2 infection initially symptomatic at the acute phase, with numerous symptoms lasting for at least two months, usually three months, from the onset of COVID-19 that cannot be explained by an alternative diagnosis” (6).

People affected with post COVID-19 sequalae are often more physically fit and younger at baseline. In addition, women are disproportionately affected by these “long haul” symptoms, with a prevalence of 64% versus 35% in men (7). The impact of long COVID on socioeconomic status is therefore significant [Chen et al. (5)]. Symptoms of possible brain origin include: loss of smell and taste; complaints of brain fog; impaired attention and memory function; sleep disturbances; pain; emotional disorders; and symptoms related to dysautonomia (breathlessness, tachycardia, orthostatic intolerance and orthostatic hypotension) (7–9).

The most debilitating symptoms include fatigue (reported in 73%) and brain fog (28%), which was self-defined as “dementia” in at least one study of 30–40-year-olds (7). Additionally, because of the varied and non-specific nature of these symptoms, many are dismissed by health care providers (10), or are simply not reported. Rehabilitation of COVID-19 survivors remains widely neglected (11) not only because of this under-recognition, but because most health care systems are still overwhelmed with acute cases. Fatigue in long-COVID is multifactorial, including ongoing hypoxia, disordered lung function, depression and chronic fatigue syndrome (11). Fatigue may improve, but this will likely depend on the etiology in each case.

The specific etiology of the cognitive symptoms remains unclear. Clinicians are increasingly aware that cognitive symptoms are not necessarily related to poor pulmonary function and dyspnoea, making their potential treatment challenging. There is evidence of direct viral spread to the central nervous system (CNS) for COVID-19 and other coronaviruses (12, 13), as well as adverse effects on the CNS from other systemic symptoms, such as hypoxia. Animal studies have shown a specific vulnerability of the hippocampus (14). If this is the case with COVID-19 infection in humans, it raises the concern of the infection having an impact on memory and possible accelerated onset of hippocampus-related neurodegenerative diseases such as Alzheimer’s dementia (AD). In addition, COVID-19 infection worsens cognitive function in those with pre-existing AD, through both direct infection effects as well as the pandemic-related social and environmental restrictions (15). The mechanism for direct infection was investigated in a post-mortem study (16). It suggests concomitant COVID-19 infection could amplify pre-existing dementia in at least two ways: (1) by modulating the expression of proteins that may worsen AD; (2) stressing the already dysfunctional neurons especially in areas with abundant hyperphosphorylated tau protein and/or β-amyloid-42; (3) potentially increasing neuroinflammation (16).

Most of the research focuses on patients requiring admission to hospital for COVID-19 infection. However, cognitive symptoms especially, can also occur in people who had a seemingly mild infection (not requiring hospitalization) from which they apparently fully recovered, i.e., their acute viral symptoms resolved (11, 13). In one study where 80% of participants had a mild COVID-19 infection, 28.6% reported new “dementia” symptoms (7).

Importantly, metabolic brain studies (17–21) have shown cerebral hypometabolism using ^18^F-fluorodeoxyglucose-positron emission tomography (FDG-PET) imaging. This research shows a consistent pattern of frontal hypometabolism and cerebellar hypermetabolism in post COVID-19 patients complaining of cognitive deterioration. FDG-PET imaging also shows cerebral glucose hypometabolism in other conditions associated with cognitive decline such as mild cognitive impairment (MCI) and AD (22). This glucose hypometabolism can potentially be countered by providing a dietary source of substrate to increase serum ketone bodies (23, 24) with, in the case of MCI, a direct and significant benefit in several cognitive domains (25).

As a therapeutic strategy, *endogenous* ketosis to correct brain glucose hypometabolism requires a significant and prolonged reduction in insulin, which is typically achieved by fasting and/or very significant reduction in carbohydrate intake. However, both ketone bodies and medium-chain fatty acids (MCFA) can also be supplied from an *exogenous* dietary source, such as medium chain triglyceride (MCT, C8, caprylic acid), without needing to change energy or macronutrient intake. Such a daily MCT supplement partially overcomes the cerebral glucose hypometabolism in MCI (25, 26) and AD (27, 28) with concomitant improvement in cognitive symptoms in the domains of memory, executive function, language, and processing speed. Some studies suggest ketone bodies selectively target neuronal mitochondrial function (29, 30). Other than the direct effect of ketone provision, MCT can also directly inhibit AMPA receptors (glutamate receptors), and change cell energetics through mitochondrial biogenesis (31).

### Hypothesis

**Figure 1:**
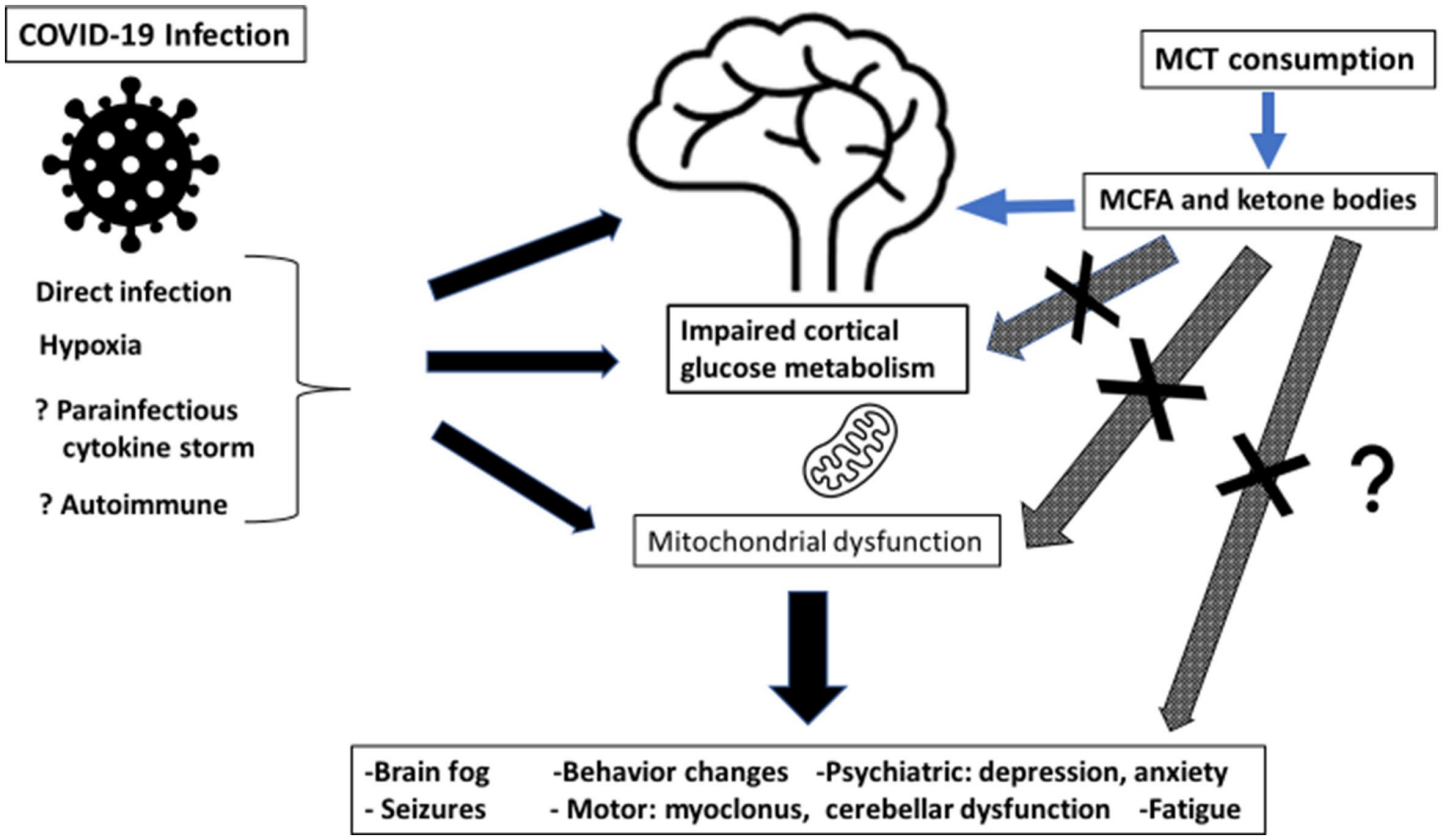
Summary of potential mechanism of action of medium chain triglyceride (MCT) consumption for post-COVID-19 cognitive symptoms. MCT, medium chain triglyceride; MCFA, medium chain fatty acids.

Ketosis induced by MCT oil supplementation will improve brain energy metabolism post-COVID-19 because ketone bodies will correct/bypass persistent brain glucose hypometabolism, resulting in better cognitive function and less brain fog. See Figure 1.

## Evaluation of the hypothesis

### Generation of ketone bodies

#### Endogenous

The brain metabolizes 120–130 g/day of glucose (32). It consumes 16% of the body’s total O_2_ consumption, despite representing only 2.0–2.3% of adult body weight. In numerous physiological states, including the neonatal period, fasting, calorie restriction, starvation, post exercise, and very low carbohydrate diets, the body is able to generate ketone bodies (acetoacetate and beta hydroxybutyrate) as an alternative brain energy source to glucose (33). *Endogenous* ketone bodies are normally generated by beta-oxidation of long chain fatty acids released from adipose tissue. This process is dependent on low insulin levels, which enhances lipolysis in white adipose tissue due to the suppressed insulin-induced inhibition of hormone sensitive lipase (34). In long-term fasting, ketone bodies can supply >60% of the brain’s energy requirements (32, 35), and are actually preferentially taken up by the brain over glucose when adequate amounts of both energy substrates are available (36–39), although glucose will always be used in conjunction with ketone bodies. *Endogenous* ketosis can also be induced with a very low carbohydrate high fat (VLCHF) diet (40) or a ketogenic diet (41).

Whether ketogenesis or catabolism is normal in COVID-19 acute illness or post-COVID is unknown. Indeed, if treatment includes intravenous dextrose, insulin will undoubtedly suppress endogenous ketone production.

#### Nutritional (exogenous)

Long term compliance with fasting or VLCHF, LCHF and ketogenic diet (KD) regimes is challenging (40). Although the cardiometabolic safety of the KD is becoming less of a concern, it is still best applied under close medical or dietetic supervision (40, 41).

**Figure 2:**
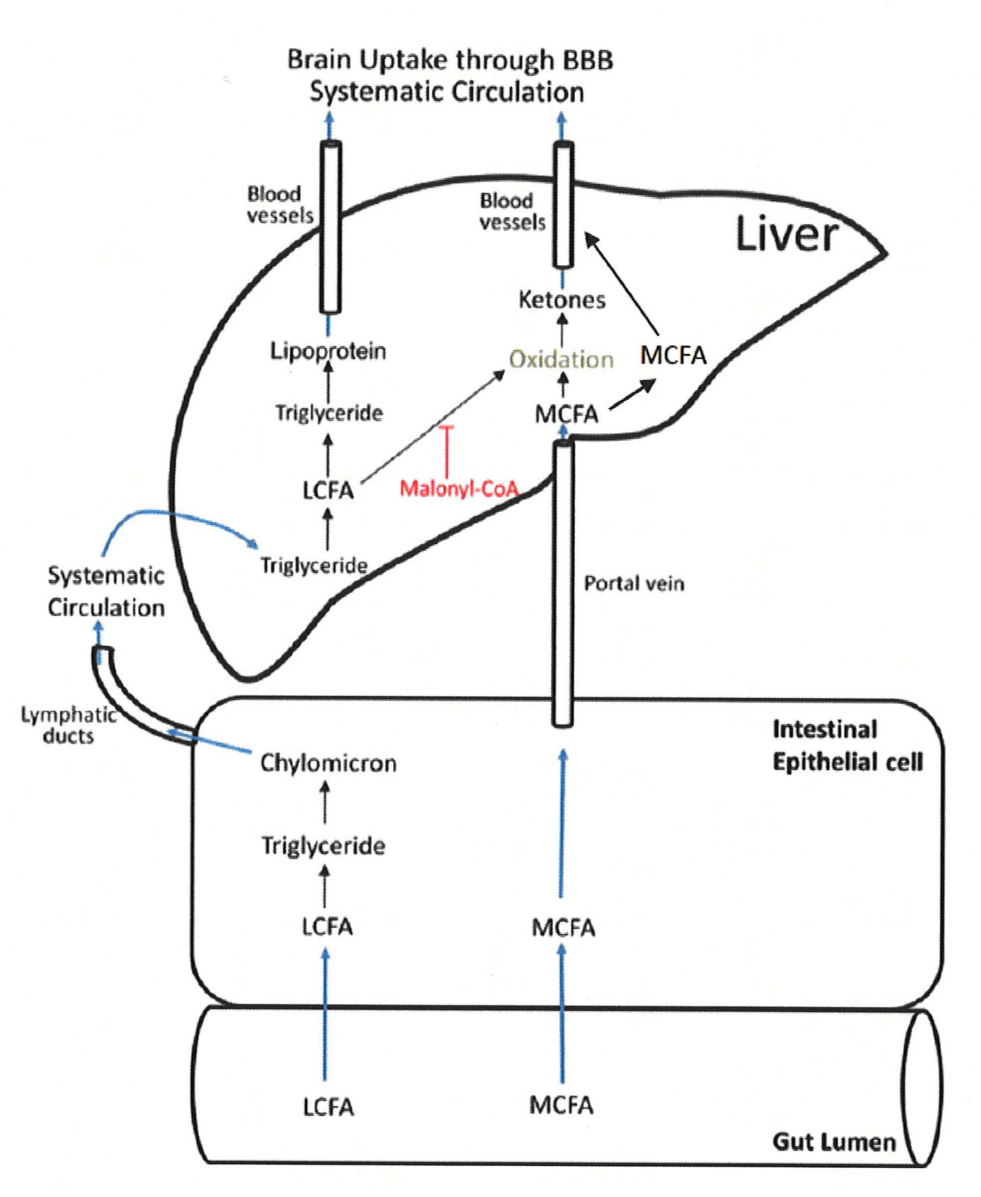
MCT absorption and metabolism. LCFA, long chain fatty acids; MCFA, medium chain fatty acids (22) (Figure adapted) (Reproduced with permission by the publisher).

MCT consumption, on the other hand, has the potential advantage of inducing nutritional ketosis without the need for a drastic change in dietary habits, especially during a time when a person is perhaps the least able to adjust (24–28, 30, 39, 42–46). Medium chain fatty acids (6-12C) from MCT are rapidly absorbed from the gastrointestinal tract, and unlike long chain fatty acids (13-22C), move directly into the liver *via* the portal vein and do not promote triglyceride synthesis (23). See Figure 2 (23). Once absorbed some are metabolized into ketone bodies, which enter the citric acid cycle to provide energy *via* adenosine triphosphate (ATP). The remainder of the absorbed MCFA enter the circulation and cross the blood brain barrier as MCFAs (24, 31, 47, 48). Unlike long-chain fatty acids, MCFAs are able to directly enter mitochondria without the need for carnitine-dependent transport. This allows for rapid Beta-oxidation and ATP generation (24, 49), which is particularly important in the role of MCT for epilepsy management (50–52). *Exogenous* ketosis from MCT is independent of the fasting state, plasma insulin or carbohydrate intake.

There is a direct dose–response relationship between MCT consumption, plasma ketone [B-hydroxybutyrate (BHB)] response (46) and brain ketone uptake (30). MCT can be consumed *per se* or emulsified into a drink, and is generally well tolerated provided the dose is slowly increased (28). Research interest is growing in the use of lipid nanoparticles as another potential mode of delivery, perhaps allowing for increased doses and better MCT tolerability (53). Reducing simple sugar intake while providing MCT may improve insulin sensitivity and potentially help endogenous ketosis (54). Ketone response can also be easily assessed with finger-prick BHB testing (46).

Long term safety with MCT consumption has been established in pediatric populations over the years with the use of MCT in pediatric supplements (55). Safety in adults with AD and MCI participants has also been shown (28, 56). MCT supplementation requires input from knowledgeable physicians, dietitians, or other health professionals, to optimize potential beneficial effects and minimize side effects.

As previously noted, some of the MCT consumed is converted into ketone bodies in the liver, but some also remains as MCFAs in the blood, the relative amount of which depends on the MCT consumed. For example, C8 MCT produces more ketone bodies than C10 MCT (47).

Ketone bodies (such as BHB) and MCFAs have been shown to be supplementary cognitive fuels in different cellular compartments in the brain (57). BHB and MCFAs enter the brain via different mechanisms: BHB depends on monocarboxylate transporters; while MCFAs appear to diffuse passively across cell membranes (50, 58). Studies in animal models suggest that MCFAs are metabolized by astrocytes, although they may also support neuronal metabolism (49). In contrast, neurons appear to be the primary cellular compartment of ketone body metabolism (58). This appears to be a non-competitive process (57). It is currently unknown whether, apart from ketone body production, MCFAs *per se* have value as auxiliary fuels to the brain (57). However, this data suggests that using MCT *per se*, rather than ketone esters or a ketogenic diet alone, may well provide auxiliary fuel to both major cell types in the central nervous system, and may be preferred in conditions where there are defects in glucose metabolism in astrocytes and neurons (57).

In epilepsy treated with MCT there is no clear correlation between serum ketone body levels and seizure reduction, suggesting that in this circumstance, at least, there is a role of the MCFA *per se* (particularly C10 MCFA) for seizure reduction (52). To date, MCT supplementation has not be assessed for cognitive effects in post COVID-19 cognitive complaints.

### Possible causes of cognitive impairment post COVID-19

**Figure 3:**
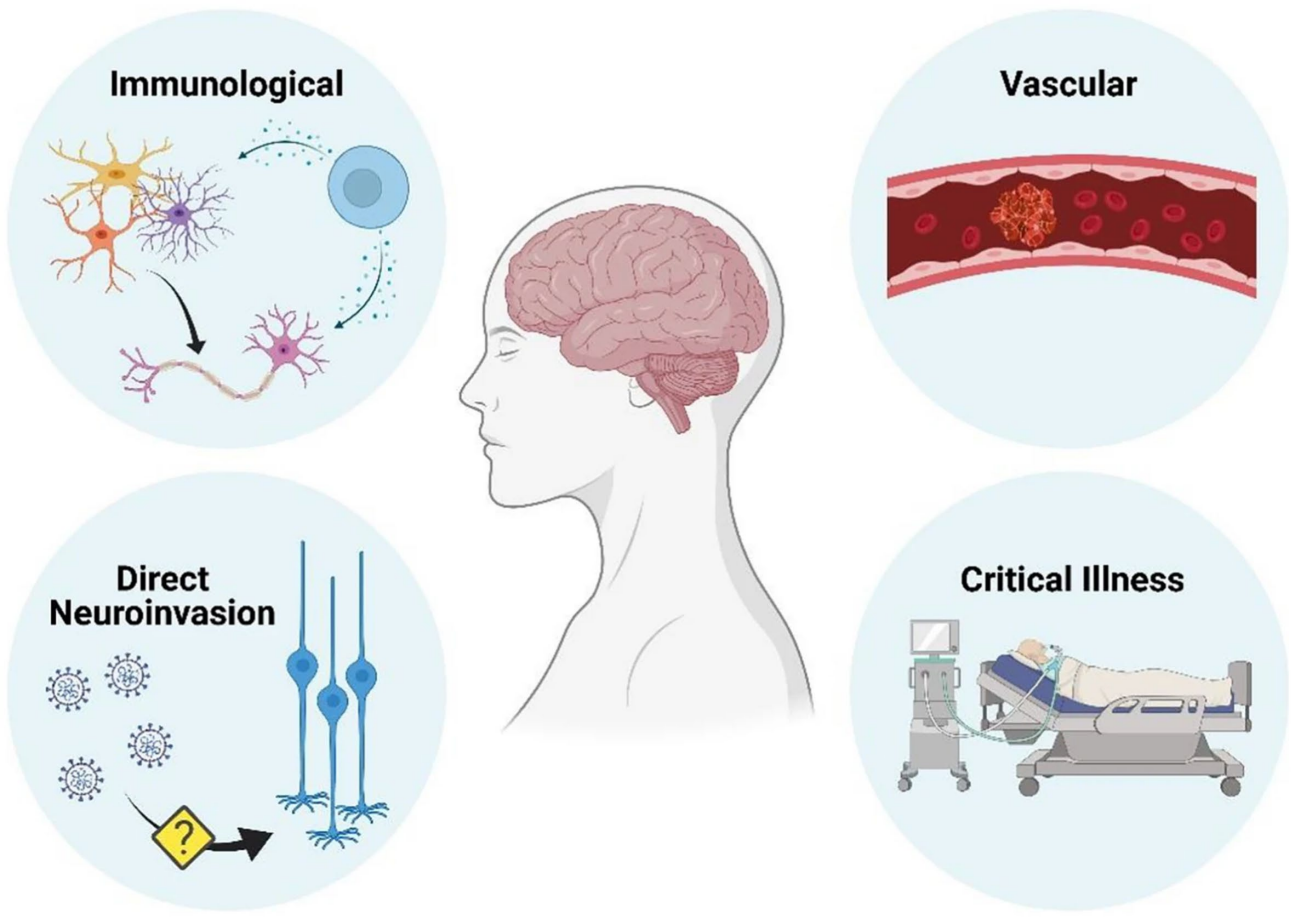
Mechanisms by which neurological disease can occur as a result of COVID-19 infection (59) (Reproduced with permission from the publisher). Figure created with BioRender.com.

The etiology of post COVID-19 cognitive symptoms continues to be investigated with both direct and indirect causes possible. See Figure 3 (59). Vascular disease appears to be disproportionately common in COVID-19 than in comparable infections, whereas immunologically mediated neurological conditions are similar in frequency (59). The evidence supporting direct central nervous system by SARS-CoV-2 as a cause of neurological disease is conflicting (59).

#### Direct

##### Direct invasion

Although direct invasion of the central nervous system (CNS) by COVID-19 has been reported it is still debated (59) One hypothesis of CNS infection through a nasopharyngeal route is supported by clinical observations of frequent and persistent anosmia/dysgeusia (12, 60, 61).

However, of note, no descriptions of viral inclusions or reactive cellular changes typical of true infection have been reported (62, 63), and there is no evidence COVID-19 can cross the blood brain barrier (59). Contradictory reports from different groups highlight the technical challenges involved, and the possibility of viral contaminants from the blood or endothelium (59).

#### Indirect

##### Immunological

###### Acute inflammation

The hyperinflammatory state secondary to COVID-19 infection, causes a massive release of cytokines and chemokines that could alter the permeability of the blood–brain barrier. This phenomenon can activate a neuroinflammation cascade (64). A COVID-19 patient presented with only mild respiratory symptoms but with encephalitis (60), and responded to steroid therapy, suggesting the neurological symptoms could have involved a cytokine-mediated hyperinflammatory response. Although there was no evidence of SARS-CoV-2 in the CSF by RT-PCR, a direct CNS infection could not be excluded (50). Since that initial report, several studies have reported delirium and encephalitis post-COVID (65, 66).

Increased IL-6 levels in the blood and CSF in some COVID patients may support a para-infectious cytokine release, postinfectious antibody-or cell-mediated immune mechanism. Cytokines can pass the blood–brain barrier, induce central inflammatory responses and influence neurotransmitter metabolism and neural plasticity (67, 68). They induce dysfunction in brain areas implicated in emotional and behavioral regulation and cognition (such as the prefrontal cortex, basal ganglia) and fear and anxiety-related regions (such as the amygdala, insula and anterior cingulate cortex) (67).

e Silva and colleagues (68) propose that tumor necrosis factor-α (TNF-α) and IL-6 are the two major cytokines upregulated in COVID-19 that directly affect brain physiology. These cytokines are also upregulated in AD and depression (67), and therefore may be responsible for the mood and cognitive changes identified in long-COVID.

###### Chronic inflammation

Chronic inflammation occurs in several neurological disorders (69). The proinflammatory response to COVID-19 may contribute to the neurological sequelae. In addition, its effect on the immune system further promotes viral propagation. The CNS effects such as brain fog may impair individual judgment, affecting behavior and compliance with COVID prevention recommendations/protocols, thereby further promoting viral propagation (29).

So far, anti-inflammatory therapy has not been shown to be useful in affecting long COVID cognitive function, including dexamethasone, and tocilizumab (70, 71).

###### Autoimmune

Female preponderance has led to speculation of a possible autoimmune basis for persistent symptoms in long-COVID patients (72, 73) with some groups showing elevated anti-nuclear antibody (ANA) titers. Hypothetically, COVID-19 could also induce, by molecular mimicry-related mechanisms, the production of antibodies against neural or glial cells, as demonstrated for HSV-1, Epstein–Barr virus, and Japanese encephalitis (62).

##### Mitochondrial damage

CNS neuronal mitochondrial function requires high oxygen levels. SARS-CoV-2 genomic and subgenomic RNA (sgRNA) transcripts hijack the host cell’s machinery (74). This viral “hijacking” of the mitochondrial genome (74, 75) results in mitochondrial dysfunction which compromises the high oxygen demands of the neurons causing cerebral hypoxia (29, 76). This selective neuronal mitochondrial targeting by SARS-CoV-2 may be an evolutionary advantage, as it causes “brain fog” and behavioral changes that favor viral propagation (29, 76).

##### Vascular

A D-dimer is a degradation product of crosslinked fibrin, and reflects ongoing activation of the hemostatic system. With COVID-19 infection D-dimer levels are known to be elevated, with or without venous thromboembolism (65) suggesting *microvascular* coagulation factor activation. Fibrin amyloid microclots have recently been identified in patients with long COVID symptoms using fluorescence microscopy (77). Failed fibrinolysis of these microclots could contribute to micro-capillary blockage and tissue hypoxia, and the diversity of symptoms of long COVID (77).

*Macrovascular* disease is well documented with an increased risk for acute cerebrovascular accidents, over and above the risks associated with immobility and dehydration (21, 78). Immunologically mediated thrombosis may also play a role, with reports of the presence of anticardiolipin and antiphospholipid antibodies, as well as lupus anticoagulant (73, 79). These are prothrombotic resulting in recurrent arterial and venous thromboembolic events (73). Endotheliitis is also a likely contributing factor to pathological clotting and cerebral vascular events, as well as effects on other organs such as lung, heart, kidney and intestine (80).

##### Brainstem dysfunction

Compared to other brain regions, the brainstem has a relatively high expression of angiotensin-converting enzyme 2 (ACE2) receptors. Brainstem involvement has been shown in post-mortem studies with detection of SARS CoV-2 RNA or proteins in 53% of patients. Indeed, a recent hypothesis paper (81) discusses persistent brainstem dysfunction in long-COVID. This may be related to direct SARS-CoV-2 invasion, or a pathological immune response, or vascular activation (81).

### Glucose and insulin metabolism in COVID-19

The severity, morbidity and mortality due to COVID-19 have been shown to be increased in those with pre-existing diabetes and obesity (82, 83), and hyperglycemia promotes severity and disease progression (84, 85). In addition, COVID-19 has accelerated the global pandemic of hyperglycemia (86). Elevated blood glucose acts synergistically with COVID-19 to inactivate angiotensin converting enzyme-2 which dysregulates glycaemic control in all those cell types that are infected by the virus (87). Obesity has been reported to be associated with a greater number of long COVID symptoms (88).

Elevated serum ketones *per se* have only been reported with acute COVID-19 infection, but not in long COVID patients. Patients, both diabetic and non-diabetic, with acute COVID-19 infection have presented with ketosis or ketoacidosis (89). Another group reported an initial increase in the ketone body 3-hydroxybutyrate on the first day of acute COVID-19 infection, suggesting a ketotic-like state. Although this was reduced over a week, it still persisted to a small extent beyond 7 days (90).

One of the consequences of inflammation is insulin and glucocorticoid receptor resistance (87). These changes result in a reduction of glucose availability to peripheral tissues and the brain (91), and are particularly relevant following recovery from the acute stage while the inflammation remains. In addition, brain glucose metabolism might be a factor in the spread of the SARS-CoV-2 virus in the brain (92).

Cerebral glucose hypometabolism has also been reported in many other viral diseases including SARS, HIV, Hepatitis C, and tick-borne encephalitis. Essentially in any viral infection associated with encephalopathy (93, 94).

### Cerebral glucose hypometabolism in COVID-19 (as assessed by FDG-PET neuroimaging)

Research groups from around the globe have investigated cerebral glucose metabolism in COVID-19, both in the acute stage and over time. Initial reports were related to hospitalized patients. From France, Helms and colleagues (21) reported on 58 hospitalized COVID-19 patients. Using magnetic resonance imaging (MRI), they demonstrated bilateral frontal hypoperfusion in 11 patients. Cani and colleagues (17) reported a case study from Italy of a 77-year-old female hospitalized and ventilated for COVID-19 infection with impaired consciousness. Her respiratory symptoms subsequently improved, but her cognitive issues did not, and an FDG-PET scan showed frontal lobe hypometabolism, with bilateral frontotemporal hypoperfusion and anterior slowing on the EEG. Subsequently, Guedj and colleagues (20) reported on two male COVID-19 patients from France. After recovery (requiring admission to intensive care), persistent cognitive complaints prompted FDG-PET imaging and showed hypometabolism in the pre/post central gyrus, thalamus/hypothalamus, cerebellum and brainstem, with brain abnormalities persisting after the remission of the infectious phase. Delorme et al. (18), also from France, reported on four patients presenting with cognitive symptoms, predominantly affecting the frontal lobe, presenting 0–12 days after their COVID-19 symptoms. All had normal MRI and CSF findings, but consistent FDG-PET frontal hypometabolism and cerebellar hypermetabolism. All improved clinically with intravenous polyvalent immunoglobulin (IVIg) or pulse corticosteroid immunotherapy.

A larger study of 29 post hospitalized subjects from Germany (95) also looked at neurological sequelae. At approximately 1-month after the acute infection, they showed pathological changes on FDG-PET in 10/15 subjects with predominantly frontoparietal hypometabolism. These changes were confirmed objectively with impaired frontoparietal scores on the Montreal Cognitive assessment (MoCA) tool.

Because many cases of post COVID-19 cognitive decline are not associated with severe infection or even hospitalization, this raises speculation that there may be other mechanisms of neurological damage in addition to viral load and infection severity, as discussed previously. Symptoms of brain fog occur more commonly in those people who do not require hospitalization given the “mildness” of their COVID-19 symptoms. It is postulated, that the FDG-PET abnormalities identified may be an indicator of astrocyte dysfunction (96–99), leading to persistent synaptic dysfunction as a potential etiology for the identified symptoms and FDG-PET hypometabolism (100).

Despite their different clinical presentations, all the patients in these trials presented with similar altered FDG-PET pattern: bilateral frontotemporal hypoperfusion and cerebellar hypermetabolism. It is important to note that this *FDG-PET pattern is very distinct*, and different from that typically seen in patients with delirium, who exhibit global cortical hypometabolism (101). FDG-PET findings are more strongly associated with clinical symptoms, disease course and status than is MRI (except for cerebrovascular events) so some authors suggest *it should be considered for the initial workup, as well as for monitoring treatment in COVID-related encephalopathy* (102, 103). A recent consensus paper from the European Association of Nuclear Medicine (EANM) neuroimaging committee not only confirms the hypometabolic profile in patients with long COVID symptoms, but they go on to recommend FDG-PET neuroimaging as a way to objectively assess brain involvement in long COVID (104). They add a caveat that the testing should be done three to six months following the initial infection, or with worsening symptoms. They also reiterate the importance of a multi-disciplinary approach to also address the non-neurological symptoms (104).

Whether brain ketone metabolism is also affected remains to be determined.

### Natural history of cognitive symptoms

#### Hospitalized patients

There are still limited data on the natural history of post COVID-19 cognitive symptoms. One recent publication (105) re-evaluated eight previously hospitalized post COVID-19 patients from Freiburg, Germany, six months after initial infection. Although there was some improvement in their MoCA score from their initial score, it was still below normal cut-offs, and in the range of MCI, with persistent deficits in visuo-constructive, executive and memory function (105). Their FDG-PET images showed some improvement, but compared to normal controls, they still had significantly more frontoparietal and temporal hypometabolism. The authors felt this slow reversal was due to ongoing subcortical peri-inflammatory processes (105).

Kas and colleagues (102) looked at FDG-PET images at baseline, 1-and 6-months post COVID-19 in seven patients. All had a consistent pattern of hypometabolism in many brain areas including the frontal cortex, anterior cingulate, insula and caudate nucleus. After six months, the majority had improved clinically, but cognitive and emotional disorders of varying severity remained, with attention/executive disabilities and anxio-depressive symptoms. In addition, there were lasting prefrontal, insular and subcortical FDG-PET abnormalities. Interestingly, while some of their patients almost returned to normal cerebral metabolism (even those with initial widespread decrease), others only partially improved or, indeed, worsened. After having first improved, one patient subsequently worsened, with clinical symptoms and a new pattern of brain hypometabolism (102). This again raises the question of an associated neurodegenerative disorder. None of these patients had SARS-CoV-2 in the CSF and/or meningitis, nor had FDG-PET anomalies limited to the olfactory gyrus that could corroborate a direct viral neuro-invasion (102).

Retrospectively evaluating their long COVID patients, Guedj and colleagues reported a larger case series of 35, showing similar abnormal FDG-PET imaging changes as reported in the acute stage, when compared to their database of healthy subjects (106). In a prospective case–control study from Italy, Sollini and colleagues (107) enrolled 13 long COVID patients, and again showed FDG-PET cerebral hypometabolism in the right parahippocampal gyrus and right thalamus compared to melanoma patients matched for age and sex (107). In their study, brain hypometabolism was correlated with current symptoms, rather than the severity of the acute infection.

Larger studies of hospitalized patients included one study of 165 subjects, without baseline cognitive symptoms. They found 40% still had ongoing neurological symptoms at the 6-month follow up (108). Older age, baseline comorbidities, and infection severity were considered to be major risk factors for ongoing neurological symptoms. Neuroimaging was however not included in this study.

Adding to the imperative to explore strategies to address these brain changes, is the concerning report that similar patterns of FDG PET brain hypometabolism are also seen in pediatric COVID-19 patients (109). It is possible that some patients may have received MCTs *via* parenteral or enteral nutrition, but currently there are no publications specifically discussing this, and whether there was any cognitive impact.

#### Non-hospitalized patients

There is a paucity of data on non-hospitalized COVID-19 patients with neurological symptoms. One report (72) reviewed 100 patients attending a Neuro-COVID-19 clinic. Their average age was 43-years, and 70% were female. The main neurological complaint (81%) was brain fog. This was associated with impaired quality of life, and worse attention and working memory cognitive scores compared to non-COVID controls. MRI and EEG studies were normal. None of their participants had FDG-PET imaging.

Recent reviews have summarized the knowledge to date of FDG-PET findings in acute and long COVID-19 (110, 111).

#### Possible exacerbation of neurodegenerative diseases

Given the ongoing nature of the pandemic, long term neurological sequelae of COVID-19 are still relatively unknown, including possible acceleration of pre-existing neurological diseases. It has been reported that FDG brain hypometabolism in the pre-frontal cortex is also present in multiple neurodegenerative disorders (such as Parkinson’s disease and AD) (24, 112, 113) and neuropsychiatric conditions (114). These changes can pre-date clinical symptoms, sometimes by years. In fact, new onset Parkinsonism has already been reported post COVID-19 infection (115). COVID-19 infection can impact pre-existing mild cognitive impairment and Alzheimer’s dementia worsening the progression of both (116).

This has generated concern about a “delayed pandemic” of neurodegenerative and neuropsychiatric disease (117, 118).

### Role of ketone bodies in cognitive function

Although glucose is the main fuel for brain function, under certain circumstances, such as prolonged fasting, ketone bodies can replace glucose as the main fuel, and provide 50–60% of the brain energy needs (119). Even if glucose availability is acutely reduced, by experimental hypoglycemia, ketone infusion or medium chain fatty acid (MCFA) ingestion preserves cognitive function, and symptoms of acute glucopenia are not observed (120, 121).

Ketone bodies are not only for brain energy metabolism; they serve as lipogenic and steroid biosynthetic substrates in many tissues including the developing brain, lactating mammary gland and liver (33). They are avidly oxidized in the heart and muscle, as an alternative and glucose-sparing fuel source, and the myocardium is the highest ketone body consumer per unit mass (33). Ketone bodies are also signaling molecules for cell-surface and intracellular receptors (33), and therefore regulate mitochondrial metabolism, energetics, and reactive oxygen species (ROS) production (33). This increases seizure threshold and is felt to be one of the mechanisms of the beneficial effects of ketone bodies in epilepsy (122). They also drive protein posttranslational modification and are modulators of inflammation and oxidative stress (123). Not surprisingly, therefore, they are being investigated for their role in cell metabolism, homeostasis, and signaling under a wide variety of physiological and pathological states (124).

Ketone bodies generated from MCT consumption not only spare glucose, but also support brain metabolism during energy crises, without prior adaptations from fasting (125). They have neuroprotective effects through two main mechanisms: improved mitochondrial function, and regulation of gene expression (126).

Cerebral insulin resistance is known to be a contributing factor in AD (127, 128). This brain insulin resistance aggravates toxic Aβ production and tau-hyperphosphorylation (129–132). The metabolism of ketone bodies mitigates some of the negative CNS effects of hyperglycemia (133), thereby improving insulin sensitivity and attenuating insulin resistance (134, 135).

### Evidence for ketone bodies in other cognitive disorders

Neuroinflammation is a common feature in neurodegenerative disease and may promote a brain energy crisis (136), and this can be mitigated by ketone bodies. MCT use in this hypothesis is not being suggested as an anti-inflammatory agent, but for its direct effect on cerebral metabolism.

In other neurocognitive disorders associated with documented FDG-PET brain hypometabolism, such as MCI and AD, patients present with a decline in their cognitive abilities ranging from subjective complaints to more objectively and clinically defined cognitive deficits. These FDG-PET identified metabolic changes can pre-date the clinical neurological deficits (39, 113). In Parkinson’s disease, amyotrophic lateral sclerosis, and Huntington disease, glucose hypometabolism in selected brain regions is prominent, and correlates with disease severity (24, 34). In addition, AD and MCI patients have changes in mood often presenting with features of depression and apathy, both of which are also seen in the post COVID-19 state.

Unlike for COVID-19 infection, there is data in AD and MCI that shows, despite impaired cerebral glucose metabolism, cerebral ketone uptake is preserved (25, 26).

Exogenous ketone bodies, supplied by MCT consumption, may have beneficial effects on cognitive outcomes in both established AD and MCI (25, 27, 28, 43–45, 137, 138). Results are sometimes conflicting, likely related to different MCT formulations, outcome measures, dose, duration, and participant inclusion criteria. A recent meta-analysis of the trials of MCT supplementation in AD by Avgerinos and colleagues, concluded that MCTs can induce mild ketosis and may improve cognition in patients with MCI and AD (139). MCT supplement doses used in clinical trials varies depending on the study design, MCT composition and formulation. There is no clear dosing regime, but data so far suggests a minimum of 28 g of MCT is needed daily to have a measurable clinical impact (25–28). Dividing the doses (ideally 3–4/day) not only improves tolerability, but also facilitates higher levels of ketone bodies and MCFA throughout the day.

Other than being a source of brain energy, in mouse models of AD, ketone bodies were reported to show cognition-sparing, and reduction of amyloid-beta and tau pathology (140, 141). This raises the intriguing possibility that MCT consumption could also potentially affect the recently described fibrin amyloid microclots felt to be contributing to symptoms in long COVID patients (77).

The effect of ketone bodies on feeding behavior, energy expenditure, mood and behavior, and neuroprotection have been reported, and are summarized in a recent review (123).

## Discussion

The consequences of COVID-19 infection on cognitive function in both the acute and long COVID phases, in previously cognitively normal patients, has now been well documented. As more patients are followed long-term, increased data will become available as to the duration, social and psychological consequences of these cognitive deficits. What remains to be seen is whether this will translate into the development of other neurodegenerative diseases such as Alzheimer’s dementia and Parkinson’s disease, and whether this will occur at an earlier age of onset than is currently seen. A “delayed pandemic” of neurodegenerative and neuropsychiatric disease is predicted (117, 118). The societal and health systems consequences of this could be catastrophic.

In addition, it is now well recognized that COVID-19 infection can impact pre-existing mild cognitive impairment and Alzheimer’s dementia, worsening the progression of both (116). It can cause delirium which may not resolve, or may unmask undiagnosed MCI.

MCT is a component of coconut oil, and readily available worldwide. Given the cerebral glucose hypometabolism documented post COVID-19, as summarized in Figure 1, we hypothesize that treatment of neurological symptoms in post COVID-19 patients using MCT supplementation will provide clinical benefit in the short term, and perhaps aid functional recovery of the brain in the long term. The fact that cerebral glucose hypometabolism has also now been documented in the pediatric COVID-19 population is particularly concerning, but it is reassuring that there has already been experience and an established safety profile with MCT supplementation in the pediatric population.

If there is improvement in symptoms with MCT supplementation, further research will need to be done to evaluate whether this is a direct effect on the cerebral glucose hypometabolism, or whether it is an indirect effect through improvement of mitochondrial function; an effect on post COVID fatigue; and/or, its anti-amyloid activity reducing the number of fibrin amyloid microclots.

To date over 500 million people have had a documented COVID-19 infection. Conservatively estimating 30% will experience long COVID symptoms, of which 30% will have cognitive complaints, leaves at least 45 million people with cognitive decline from their baseline. Any strategies to aid in cognitive recovery, or mitigate the cognitive effects of the disease will have significant consequences at personal, population and health system levels.

## Data availability statement

The original contributions presented in the study are included in the article/supplementary material, further inquiries can be directed to the corresponding author.

## Author contributions

All authors listed have made a substantial, direct, and intellectual contribution to the work and approved it for publication.

## Conflict of interest

SC has consulted for or received travel honoraria or test products from Nestlé Health Science, Abbott, and Cargill.

The remaining authors declare that the research was conducted in the absence of any commercial or financial relationships that could be construed as a potential conflict of interest.

## Publisher’s note

All claims expressed in this article are solely those of the authors and do not necessarily represent those of their affiliated organizations, or those of the publisher, the editors and the reviewers. Any product that may be evaluated in this article, or claim that may be made by its manufacturer, is not guaranteed or endorsed by the publisher.

## Footnotes

^1^www.worldometers.info/coronavirus/
